# Impact of Social Determinants of Health on Melanoma Nodal Surveillance in a Multi-institutional Cohort

**DOI:** 10.1245/s10434-024-16498-w

**Published:** 2024-11-22

**Authors:** Kelsey B. Montgomery, M. Chandler McLeod, Danielle K. DePalo, Michelle M. Dugan, Jonathan S. Zager, Kelly M. Elleson, Michael S. Sabel, Tina J. Hieken, Lisa A. Kottschade, David W. Ollila, Veronica Pham, Dion Archer, Russell S. Berman, Ann Y. Lee, Jessica A. Cintolo-Gonzalez, Hannah G. McDonald, Sydney Winchester, Erin E. Burke, Kristen E. Rhodin, Georgia M. Beasley, Kristy K. Broman

**Affiliations:** 1 Department of Surgery, University of Alabama at Birmingham, Birmingham, AL USA; 2 Department of Cutaneous Oncology, Moffitt Cancer Center, Tampa, FL USA; 3 Department of Surgery, University of Michigan, Ann Arbor, MI USA; 4 Department of Surgery, Mayo Clinic, Rochester, MN USA; 5 Department of Surgery, University of North Carolina, Chapel Hill, NC USA; 6 Department of Surgery, NYU Langone Health, New York, NY USA; 7 Department of Surgery, University of Vermont, Burlington, VT USA; 8 Department of Surgery, University of Kentucky, Lexington, KY USA; 9 Department of Surgery, Ohio State University, Columbus, OH USA; 10 Department of Surgery, Duke University Medical Center, Durham, NC USA; 11 Institute for Cancer Outcomes and Survivorship, University of Alabama at Birmingham, Birmingham, AL USA; 12 Department of Veterans Affairs, Birmingham VA Medical Center, Birmingham, AL USA

**Keywords:** Melanoma, Sentinel node positive, Nodal surveillance, Social determinants of health

## Abstract

**Background:**

Nodal surveillance (NS) has overtaken completion lymphadenectomy as the preferred management for sentinel node-positive (SLN+) melanoma, but requires frequent exams and nodal ultrasound (US). Social determinants of health (SDoH) may affect US adherence in real-world populations, and evaluation of these potential impacts is needed.

**Methods:**

Adults with SLN+ melanoma diagnosed from July 2017 to December 2019 who received NS at nine cancer centers were identified retrospectively. Exposures included insurance status, travel distance, and Centers for Disease Control and Prevention (CDC) Social Vulnerability Index (SVI), a validated measure of area-level SDoH, indicated as 0 (low) to 1 (high) vulnerability. The primary outcome was US adherence (≥ 1 study per 6-month follow-up interval). The secondary outcomes were combined-modality adherence [US, computed tomography (CT), or positron emission tomography (PET)] and loss to follow-up (LTFU). Bivariate analyses and mixed-effects multivariable logistic regression were performed.

**Results:**

Most of the 519 patients were male (57%), non-Hispanic white (94.4%), and insured privately (45.3%) or by Medicare (43.5%). The median travel distance was 63.3 miles (interquartile range [IQR], 31.2–111.0 miles), and the median SVI was 0.426 (IQR, 0.253–0.610). The surveillance adherence rates were 41.6% for US and 75.1% for combined modalities. No significant differences in US adherence were observed based on sociodemographic covariates in regression analysis. Medicaid (odds ratio [OR], 3.12; *p *= 0.02) and uninsured (OR 4.48; *p *= 0.01) patients had increased likelihood of LTFU.

**Conclusions:**

Less than half of the patients in this multicenter cohort achieved US adherence, although the rates improved with combined modalities. Medicaid or non-insurance were social risk factors for LTFU. Optimizing surveillance practices for socially vulnerable groups will be crucial for the ongoing real-world implementation of NS.

**Supplementary Information:**

The online version contains supplementary material available at 10.1245/s10434-024-16498-w.

After the publication of results from the Second Multicenter Selective Lymphadenectomy Trial (MSLT-II) in June 2017 and the German Dermatologic Cooperative Oncology Group Selective Lymphadenectomy Trial (DeCOG-SLT) in 2016, nodal surveillance for sentinel lymph node-positive (SLN+) cutaneous melanoma has been widely adopted, with a recent multicenter study citing a nodal surveillance rate of 84% among a large post-MSLT-II SLN+ cohort.^1–3^ Nodal surveillance in the MSLT-II protocol entailed nodal ultrasound (US) and physical examination every 4 months for the first 2 years after sentinel lymph node biopsy (SLNB), then every 6 months for the subsequent 3 years. Compared with completion lymph node dissection (CLND), this frequent surveillance strategy combined with timely therapeutic lymph node dissection for nodal recurrences demonstrated equivalent melanoma-specific survival among the MSLT-II trial cohort.

In real-world populations, however, pragmatic challenges to nodal surveillance adherence are likely to arise that may be impacted by social determinants of health (SDoH), the conditions in which people live, work, and age.^4^ Findings have shown that SDoH influences a wide variety of health outcomes including cancer care and surgical outcomes,^5–9^ but its influence on nodal surveillance among SLN+ melanoma patients has not previously been studied. Social risk factors such as lack of health insurance or access to reliable transportation could place SLN+ patients at risk for unsalvageable nodal recurrence if they are unable to complete regular nodal surveillance.

Given the rapid adoption of nodal surveillance after MSLT-II and DeCOG-SLT, evaluating current US surveillance adherence rates and identifying potential barriers to consistent nodal surveillance among SLN+ melanoma patients, including social risk factors, has become a critical need to optimize real-world surveillance practices. Therefore, this study aimed to evaluate the impact of individual- and area-level SDoH on adherence to nodal surveillance for SLN+ melanoma patients in a multi-institutional cohort across nine tertiary cancer centers in the United States.

## Methods

### Study Overview

This study was approved by the Institutional Review Board of the University of Alabama at Birmingham, which served as the coordinating center. Participating centers (listed in Table S1) obtained approval from their respective review boards, and data use agreements were executed between the coordinating and all participating centers before data transfer. Participating centers were recruited from United States academic cancer centers and constituents of the International High-Risk Melanoma Consortium, a collaborative group of 21 melanoma centers from the United States, Europe, and Australia. The guidelines of Strengthening the Reporting of Observational Studies (STROBE) for the reporting of observational studies were used.^10^

### Cohort Selection and Data Sources

The study enrolled patients with SLN+ cutaneous melanoma who underwent SLNB at a participating center between July 2017 and December 2019. Only patients undergoing nodal surveillance were included. Therefore, the study excluded those who underwent immediate CLND or had distant metastases detected on postoperative staging imaging (within 3 months after SLNB). To allow for evaluation of area-level SDoH using the CDC/ASTDR Social Vulnerability Index (SVI),^11^ one patient who did not have a United States address of residence was excluded.

Each participating site performed retrospective abstraction of data collected during routine clinical care at its center, including sociodemographic, disease, treatment, and surveillance variables. Surveillance imaging use was abstracted by counting the total number of surveillance US, computed tomography (CT), and positron emission tomography (PET) studies performed between the date of SLNB and the date of recurrence (for the patients who experienced disease recurrence) or the date of last clinical contact (for patients who did not experience disease recurrence). Surveillance imaging studies completed at outside facilities were included in these totals if documentation was available to the participating center to confirm surveillance imaging completion. One participating center did not have CT or PET data available at the time of analysis, so no combined-modality adherence was calculated for this center.

Individual-level SDoH variables included insurance payor and travel distance from the patient’s home address to the treating center. The CDC/ASTDR SVI was selected as a measure of area-level SDoH.^11^ This index uses 16 variables from United States Census data to rank every census tract by the level of social vulnerability, from 0 (lowest vulnerability) to 1 (highest vulnerability). Together with an overall SVI value per census tract, the publicly available SVI dataset includes census tract-level values for four SVI themes (i.e., component subgroups of overall SVI): theme 1 (socioeconomic status), theme 2 (household characteristics), theme 3 (racial and ethnic minority status), and theme 4 (housing type and transportation).^11^ Although SVI was originally designed for emergency response planning, it has been widely used in clinical outcomes research as a proxy measure for area-level SDoH.

For this study, the smallest geographic unit available from all the participating centers was the zip code. Therefore, a census tract-to-zip code crosswalk dataset from the United States Department of Housing and Urban Development and United States Postal Service^12^ was used to convert census tract-level SVI to a zip code-level SVI, which was calculated using the median SVI value across all census tracts within a given zip code. These zip code-level SVI data then were linked to patient-level clinical data using patient zip code of residence.

### Study Outcomes and Definitions

The primary study outcome was US adherence, defined using a ratio of the number of surveillance US studies to the 6-month follow-up intervals, with a ratio of 1 or greater considered to indicate US-adherent status. Follow-up intervals were calculated from the date of SLNB to the last date of clinical contact or disease recurrence, as applicable. Although MSLT-II surveillance protocols recommend nodal US every 4 months for the first 2 years after SLNB followed by US in 6-month intervals for the subsequent 3 years, a uniform 6-month follow-up interval was chosen to allow for some flexibility in the timing of US study completion during this early post-MSLT-II implementation period.

The secondary study outcomes were adherence to the combined surveillance imaging modality (1 or more US, CT, and/or PET studies per 6-month follow-up interval) and loss to follow-up evaluation (LTFU). Combined-modality adherence was chosen as a secondary outcome to account for potential differences in surveillance imaging preferences between centers, particularly among patients receiving adjuvant systemic therapy. For the purposes of this study, LTFU was defined as no clinical contact for at least 12 months during the surveillance period after SLNB. This was selected as an additional secondary outcome given the importance of regular clinical evaluation to identify possible disease recurrence after SLNB.

### Statistical Analysis

Descriptive statistics were calculated, with results reported as medians with interquartile ranges (IQRs) for continuous variables and frequencies with percentages for categorical variables. Bivariate analyses using Pearson’s chi-square tests, Fisher’s exact tests, and Wilcoxon rank-sum tests were performed to compare US-adherent and US-nonadherent groups. Mixed-effects multivariable logistic regression models were created for each primary and secondary outcome with treating centers as random effects to account for center-level clustering. Covariates included patient age, sex, travel distance (< 50, 50–100, or > 100 miles); overall SVI quartile; insurance payor; receipt of adjuvant systemic therapy; and treatment at a prior MSLT-II participating site. Intraclass correlation coefficients (ICCs), which describe the proportion of variation in the outcome of interest (i.e., US adherence) attributable to group-level effects (i.e., treatment center effects) were calculated for each model.

Secondary analyses were designed to evaluate the association of each of the four SVI themes with US adherence outcomes. Mixed-effects multivariable logistic regression models were created for each SVI theme, with the same covariates as the primary analysis except for SVI theme quartiles instead of overall SVI quartiles. Center-level results are reported using a randomly assigned alphabetical letter to protect the confidentiality of the participating centers and their patients. The alpha level for all statistical tests was set at 0.05. Statistical analyses were conducted in R version 4.3.0 (R Core Team).^13^

## Results

### Descriptive Statistics

Cohort demographics are detailed in Table 1. Among an overall cohort of 519 patients across nine participating centers, the median age was 61 years (IQR, 48.0–71.0 years). A majority of the patients were male (296, 57%), non-Hispanic white (488, 94%), and insured through private insurance (*n *= 235, 45.3%) or Medicare (*n *= 226, 43.5%). Less than 10% of the patients were insured by Medicaid (*n *= 27, 5.2%) or not insured (*n *= 17, 3.3%).

**Table 1:** Cohort demographics

Characteristic	(*n *= 519) *n* (%)
Median age: years (IQR)	61.0 (48.0–71.0)
Male sex	296 (57.0)
Race/ethnicity	
Non-Hispanic white	488 (94.0)
Hispanic	11 (2.1)
Other or unknown	10 (1.9)
Non-Hispanic black or African American	4 (0.8)
Asian	4 (0.8)
American Indian/Alaska Native	2 (0.4)
Insurance	
Private	235 (45.3)
Medicare	226 (43.5)
Medicaid	27 (5.2)
Uninsured	17 (3.3)
Other government insurance	7 (1.3)
Unknown	7 (1.3)
Median travel distance: miles (IQR)	63.3 (31.2–111.0)
Median overall SVI (IQR)	0.426 (0.253–0.610)
Primary tumor location	
Head/neck	63 (12.1)
Upper extremity	94 (18.1)
Trunk	219 (42.2)
Lower extremity	143 (27.6)
Median Breslow depth: mm (IQR)	2.0 (1.2, 3.6)
AJCC pathologic stage	
IIIA	202 (38.9)
IIIB	101 (19.5)
IIIC	210 (40.5)
IIID	5 (1.0)
Received adjuvant systemic therapy	224 (43.2)
Median Follow-up: months (IQR)	53.4 (42.3–62.2)
Lost to follow-up	151 (29.1)
Prior MSLT-II study site	289 (55.7)

IQR, interquartile range; SVI, Social Vulnerability Index; AJCC, American Joint Committee on Cancer 8th edition; MSLT-II, Second Multicenter Selective Lymphadenectomy Trial

Variables are represented as median (IQR) for continuous measures and frequency (%of total cohort) for categorical measures.

**Fig. 1:**
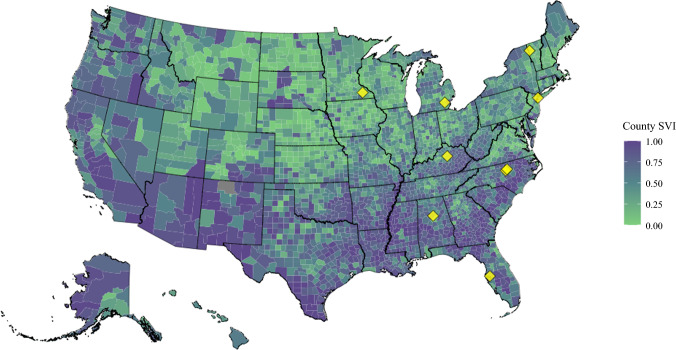
Map of county-level CDC Social Vulnerability Index (SVI) values and participating centers. SVI ranges from low vulnerability (0: *green*) to high vulnerability (1: *purple*). The participating centers are indicated by yellow diamonds. CDC, Centers for Disease Control and Prevention

The median travel distance was 63.3 miles (IQR, 31.2–111.0 miles), although this varied widely by institution (range of center-level median travel distance, 10.7–101.5 miles; *p *< 0.001). The median overall SVI was 0.426 (IQR, 0.253–0.610), favoring lower social vulnerability, but this again varied widely by institution (range of center-level median SVI, 0.302–0.559; *p *< 0.001). A map of county-level overall SVI and the location of participating centers are depicted in Fig. 1.

Most primary tumors were located on the trunk (*n *= 219, 42.2%) or lower extremity (*n *= 143, 27.6%), with a median Breslow depth of 2.0 mm (IQR, 1.2–3.6 mm). Among the overall cohort, 224 patients (43.2%) received adjuvant systemic therapy, with a range of 7.1–95.0% across treating centers. The median follow-up period was 53.4 months (IQR, 42.3–62.2 months), with 151 patients (29.1%) lost to follow-up at some point during their surveillance period. Among the overall cohort of 519 patients, 445 (85.7%) had at least one 6-month follow up interval for analysis of surveillance imaging adherence.

### Adherence to Surveillance Ultrasound

**Fig. 2:**
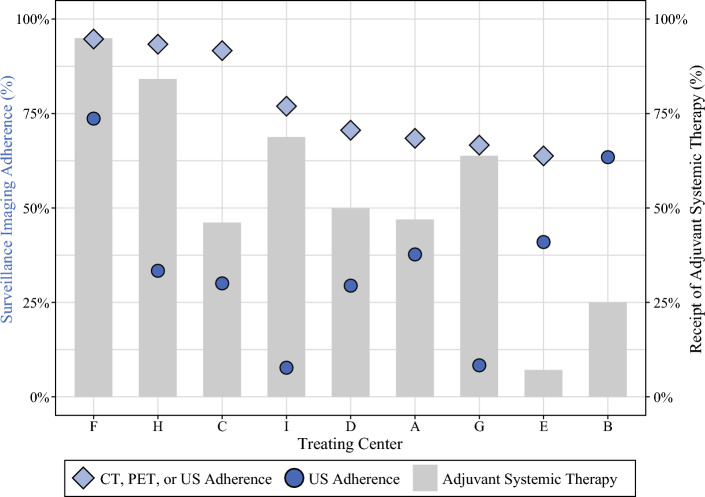
Treating center-level variation in adherence to ultrasound (US: *dark blue circles*), combined surveillance imaging modalities (US, CT, or PET: *light blue diamonds*), and proportion of patients receiving adjuvant systemic therapy (*gray bars*). Treating centers are ordered left to right from highest to lowest combined-modality adherence percentage. *Note*: CT/PET surveillance imaging data were not available for treating center B, so no combined adherence percentage was calculated. US, ultrasound; CT, computed tomography; PET, positron emission tomography

**Fig. 3:**
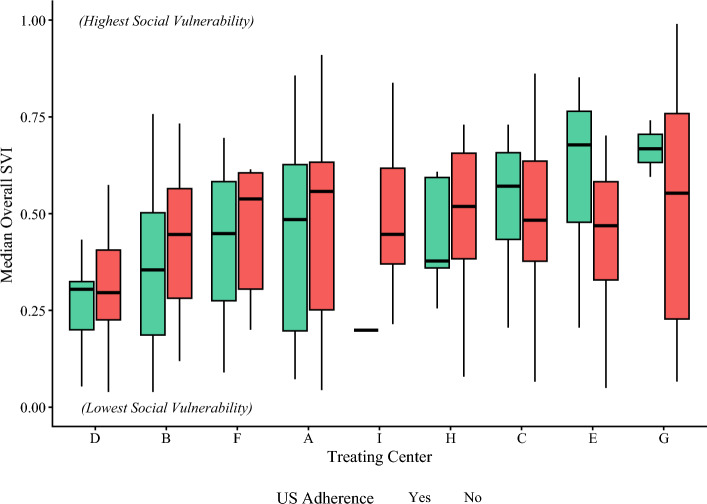
Comparison of median SVI among patients at each treating center stratified by nodal surveillance ultrasound (US) adherence. Boxplots depict the SVI values for adherent (*green boxplots*) versus nonadherent (*red boxplots*) groups at each center, with the median value represented by the bold horizontal line, the box representing the IQR, and whiskers representing the range. Treating centers are ordered from left to right by lowest to highest median overall SVI: 0 (low social vulnerability), 1 (high social vulnerability). *Note*: There is no boxplot for the US-adherent cohort from treating center 1 as there was only one patient in that subgroup. SVI, Social Vulnerability Index; IQR, interquartile range

Adherence to surveillance US among the overall cohort was 41.6% among the patients who had at least one 6-month follow-up interval (185 of 445 patients). Ultrasound adherence varied widely among treating centers, ranging from 7.7 to 73.7%, as depicted in Fig. 2. The comparison of sociodemographic and treatment characteristics between the US-adherent and US-nonadherent groups showed no statistically significant differences based on patient age, sex, insurance payor, travel distance, or overall SVI (Table 2). The ultrasound-adherent patients had lower social vulnerability for SVI theme 4 (housing type and transportation; 0.397 [IQR, 0.265–0.573] vs. 0.441 [IQR, 0.321–0.639]; *p *< 0.01), less frequent receipt of adjuvant systemic therapy (35.7% vs. 48.5%; *p *< 0.01), and a larger proportion patients treated at prior MSLT-II study sites (64.9% vs. 51.5%; *p *< 0.01). When each treating center’s cohort was stratified by US adherence, four of the nine centers had higher median social vulnerability among their US-nonadherent group (Fig. 3). This trend was more prevalent among the centers with a lower overall median social vulnerability.

**Table 2:** Comparison of patient characteristics by ultrasound (US) adherence and combined-modality (US, CT, or PET) adherence

Characteristic	US adherence			Combined adherence		
	Yes (*n *= 185) *n* (%)	No (*n *= 260) *n* (%)	*p* Value	Yes (*n *= 251) *n* (%)	No (*n *= 83) *n* (%)	*p* Value
Median age: years (IQR)	61.0 (48.0–69.0)	61.0 (48.0–71.0)	0.72	62.0 (47.5–70.0)	62.0 (47.0–72.0)	0.61
Male sex	102 (55.1)	151 (58.1)	0.54	141 (56.2)	45 (54.2)	0.76
Insurance						
Private	89 (48.1)	116 (44.6)	0.67	118 (47.0)	29 (34.9)	<0.01
Medicare	82 (44.3)	112 (43.1)		113 (45.0)	36 (43.4)	
Medicaid	6 (3.2)	15 (5.8)		11 (4.4)	6 (7.2)	
Uninsured	4 (2.2)	10 (3.8)		4 (1.6)	7 (8.4)	
Other government	2 (1.1)	5 (1.9)		3 (1.2)	3 (3.6)	
Unknown	2 (1.1)	2 (0.8)		2 (0.8)	2 (2.4)	
Median travel distance: miles (IQR)	62.8 (33.8–104.9)	59.7 (27.1–111.0)	0.63	58.9 (23.3–106.5)	55.2 (33.5–102.0)	0.81
Overall SVI: median (IQR)	0.415 (0.235–0.605)	0.453 (0.272–0.627)	0.21	0.460 (0.263–0.633)	0.488 (0.298–0.635)	0.63
SVI theme 1 (socioeconomic status): median (IQR)	0.455 (0.252–0.652)	0.484 (0.277–0.659)	0.47	0.502 (0.281–0.667)	0.479 (0.290–0.638)	0.56
SVI theme 2: (household characteristics): median (IQR)	0.517 (0.336–0.724)	0.557 (0.370–0.694)	0.73	0.541 (0.340–0.711)	0.546 (0.400–0.681)	0.96
SVI theme 3: (racial & ethnic minority status): median (IQR)	0.347 (0.184– 0.471)	0.345 (0.223–0.509)	0.26	0.362 (0.225–0.508)	0.416 (0.257–0.548)	0.11
SVI theme 4: (housing type & transportation): median (IQR)	0.397 (0.265–0.573)	0.441 (0.321–0.639)	<0.01	0.445 (0.315–0.606)	0.471 (0.331–0.664)	0.24
Received adjuvant systemic therapy	66 (35.7)	126 (48.5)	<0.01	140 (56.0)	24 (28.9)	<0.001
Prior MSLT-II site	120 (64.9)	134 (51.5)	<0.01	99 (39.4)	44 (53.0)	0.03

CT, computed tomography; PET, positron emission tomography; IQR, interquartile range; SVI, Social Vulnerability Index; MSLT-II, Second Multicenter Selective Lymphadenectomy Trial

Data are presented as median (IQR) or frequency (%). Social Vulnerability Index (SVI) values range from 0 (lowest vulnerability) to 1 (highest vulnerability).

In the mixed-effects multivariable model for US adherence, none of the aforementioned sociodemographic or treatment covariates had a statistically significant association with likelihood of US adherence, although the receipt of adjuvant systemic therapy approached significance, with an odds ratio [OR] of 0.65 (95% confidence interval [CI] 0.41–1.02; *p *= 0.06). The ICC for this US adherence model was 0.26, representing the proportion of variation in US adherence attributable to treating center-level effects in the model.

### Adherence to Combined Surveillance Imaging Modalities

For combined surveillance modalities (US, CT, and/or PET), the adherence rate was 75.1% for the patients who had at least one 6-month follow-up interval and available US, CT, and PET data (251 of 334 patients). Similar to US adherence, the range of combined-modality adherence varied across treating centers, ranging from 64.1 to 94.7%. Among some centers with lower US adherence rates, the combined-modality adherence was significantly higher and similar to the center’s percentage of patients who received adjuvant systemic therapy (e.g. centers H, I, and G in Fig. 2). For example, at center H, only 33.3% of the patients received at least one surveillance US per 6-month follow-up interval, but 76.9% of the patients received at least one surveillance US, CT, or PET scan per follow-up interval, with 68.8% of the center’s cohort having received adjuvant systemic therapy.

Comparison of the combined-modality-adherent and modality-nonadherent groups, the adherent cohort had a higher proportion of privately insured patients (47.0% vs. 34.9%) and fewer patients insured by Medicaid (4.4% vs. 7.2%) or uninsured (1.6% vs. 8.4%) (*p *= 0.007 for insurance categories; Table 2). Additionally, the adherent group had a higher proportion of patients who received adjuvant systemic therapy (56.0% vs. 28.9%; *p* < 0.001) and fewer patients treated at a prior MSLT-II study site (39.4% vs. 53.0%; *p *= 0.03) than the nonadherent group. There were no statistically significant differences in overall SVI, SVI themes, travel distance, age, or sex between the groups.

In the multivariable analysis, the only statistically significant associations were a decreased likelihood of combined-modality adherence among the patients insured by Medicaid (OR 0.25; 95% CI 0.07–0.86; *p *= 0.03) and the uninsured patients (OR 0.13; 95% CI 0.03–0.54; *p *< 0.01) and an increased likelihood of adherence among those who received adjuvant systemic therapy (OR 3.14; 95% CI 1.71–5.77; *p *< 0.001) (Table 3). The ICC for center-level effects in this combined-modality-adherence model was 0.05.

**Table 3:** Mixed-effects multivariable logistic regression models evaluating likelihood of surveillance US adherence, combined-modality adherence, and loss to follow-up

Covariate (referent)	US adherence^a^		Combined adherence^a^		Loss to follow-up^a^	
	OR (95% CI)	*p* Value	OR (95% CI)	*p* Value	OR (95% CI)	*p* Value
Age (years)	0.99 (0.97–1.01)	0.19	1.00 (0.98–1.03)	0.91	1.01 (0.99–1.03)	0.22
Male sex (female)	0.79 (0.52–1.21)	0.28	1.07 (0.61–1.88)	0.81	1.08 (0.70–1.67)	0.71
Travel distance (<50 miles)						
50–100 miles	1.21 (0.72–2.02)	0.47	1.06 (0.54–2.07)	0.87	1.29 (0.77–2.18)	0.33
>100 miles	0.87 (0.52–1.45)	0.59	1.51 (0.76–3.01)	0.24	1.05 (0.61–1.78)	0.87
SVI quartile (lowest vulnerability)						
Mod low vulnerability	1.15 (0.63–2.11)	0.64	0.86 (0.37–2.00)	0.73	1.20 (0.63–2.28)	0.58
Mod high vulnerability	0.79 (0.44–1.44)	0.44	1.18 (0.52–2.70)	0.69	1.52 (0.82–2.82)	0.18
Highest vulnerability	0.97 (0.53–1.78)	0.92	0.81 (0.37–1.78)	0.61	1.41 (0.76–2.62)	0.28
Insurance (private)						
Medicare	1.22 (0.66–2.24)	0.52	0.80 (0.36–1.78)	0.59	0.67 (0.37–1.24)	0.21
Medicaid	0.48 (0.16–1.47)	0.20	0.25 (0.07–0.86)	0.03	2.99 (1.15–7.76)	0.03
Uninsured	0.50 (0.14–1.76)	0.28	0.13 (0.03–0.54)	<0.01	4.07 (1.28–12.97)	0.02
Other government	0.77 (0.13–4.67)	0.77	0.32 (0.05–1.96)	0.22	2.23 (0.43–11.65)	0.34
Unknown	2.74 (0.19–38.75)	0.45	0.37 (0.02–5.88)	0.48	0.85 (0.07–9.69)	0.89
Received adjuvant systemic therapy (no)	0.65 (0.41–1.02)	0.06	3.14 (1.71–5.77)	<0.001	0.84 (0.53–1.33)	0.46
Prior MSLT-II Site (no)	0.89 (0.17–4.68)	0.89	0.44 (0.17–1.17)	0.10	1.52 (0.33–7.00)	0.59

OR, odds ratio; CI, confidence interval; SVI, Social Vulnerability Index; Mod, moderately; MSLT-II, Second Multicenter Selective Lymphadenectomy Trial

Intraclass correlation coefficients for the multivariable regression models were 0.26 for US adherence, 0.05 for combined adherence, and 0.23 for loss to follow-up.

^a^Treating center is included as random effects to account for center-level clustering in model outcome.

### Loss to Follow-Up Evaluation

Overall, 151 patients (29.1%) were lost to follow-up evaluation during the study period, with a range of 0.0–60.2% across the participating centers. The LTFU group had higher social vulnerability in terms of overall SVI, SVI theme 1 (socioeconomic status), and SVI theme 2 (household characteristics) than the non-LTFU group, as detailed in Table 4. Additionally, the LTFU group had higher proportions of patients insured by Medicaid (7.9% vs. 4.1%) or uninsured (6.6% vs. 1.9%) (*p *= 0.03 across insurance categories). There were no statistically significant differences based on age, sex, travel distance, receipt of adjuvant systemic therapy, treatment at a prior MSLT-II study site, or SVI theme 3 or 4.

**Table 4:** Comparison of patient sociodemographic and treatment characteristics by loss to follow-up status

Characteristic	Lost to follow-up (*n *= 151) *n* (%)	No loss to follow-up (*n *= 368) *n* (%)	*p* Value
Median age: years (IQR)	60.0 (48.0–72.0)	62.0 (48.8–70.0)	0.97
Male sex	89 (58.9)	207 (56.3)	0.57
Insurance			
Private	66 (43.7)	169 (45.9)	0.03
Medicare	56 (37.1)	168 (45.7)	
Medicaid	12 (7.9)	15 (4.1)	
Uninsured	10 (6.6)	7 (1.9)	
Other government	3 (2.0)	4 (1.1)	
Unknown	2 (1.3)	5 (1.4)	
Median travel distance: miles (IQR)	67.4 (35.7–108.4)	61.9 (27.5–111.0)	0.21
Overall SVI: median (IQR)	0.515 (0.300–0.635)	0.396 (0.228–0.590)	<0.01
SVI theme 1 (socioeconomic status): median (IQR)	0.554 (0.347–0.707)	0.427 (0.248–0.639)	<0.001
SVI theme 2: (household characteristics): median (IQR)	0.604 (0.402–0.734)	0.507 (0.336–0.686)	<0.01
SVI Theme 3: (racial & ethnic minority status): median (IQR)	0.354 (0.245–0.475)	0.329 (0.179–0.484)	0.39
SVI Theme 4: (housing type & transportation): median (IQR)	0.424 (0.315–0.594)	0.418 (0.287–0.596)	0.62
Received adjuvant systemic therapy	60 (39.7)	164 (44.6)	0.66
Prior MSLT-II site	82 (54.3)	207 (56.3)	0.69

IQR, interquartile range; SVI, Social Vulnerability Index; MSLT-II, Second Multicenter Selective Lymphadenectomy Trial

Social Vulnerability Index (SVI) values range from 0 (lowest vulnerability) to 1 (highest vulnerability)

In the multivariable analysis, insurance by Medicaid (OR 2.99; 95% CI 1.15–7.76; *p *= 0.03) and uninsured status (OR 4.07; 95% CI 1.28–12.97; *p *= 0.02) were associated with increased likelihood of LTFU, with no other statistically significant associations observed among the other covariates (Table 3). The ICC for center-level effects in this model was 0.23.

### SVI Themes: Secondary Analysis

A secondary analysis was performed to evaluate the association between SVI theme subscores and the likelihood of US adherence. As detailed in Table S2, no clear trends for decreased US adherence were found, with increasing vulnerability across the four SVI themes, although there was a statistically significant decreased likelihood of US adherence among the patients in the moderate high vulnerability quartile for SVI theme 2 (household characteristics; OR 0.40; 95% CI 0.21–0.76; *p *= 0.005).

## Discussion

In this multicenter retrospective observational study evaluating melanoma nodal surveillance adherence among nine tertiary academic cancer centers, less than half of the SLN+ patients underwent regular surveillance US across 6-month follow-up intervals. When cross-sectional imaging studies were included, the combined-modality surveillance adherence increased to three fourths of the cohort. No statistically significant differences in US adherence were seen based on individual- or area-level SDoH, but lack of insurance was associated with a decreased likelihood of combined-modality adherence, and lack of insurance and insurance by Medicaid were associated with increased odds of LTFU. Overall, significant variability in use of US, cross-sectional imaging, and adjuvant systemic therapy was seen across treating centers, with center-level effects having a significant impact on variation in US adherence and LTFU outcomes.

Prior work has demonstrated variable adherence rates for nodal surveillance US among other post-MSLT-II SLN+ cohorts in the United States and internationally, ranging from 34 to 96%.^3,14–16^ In this study, US rates were lower than expected, but in some centers had an inverse association with CT or PET adherence, suggesting that surgical oncologist or institutional preferences for cross-sectional modalities versus US may have influenced this finding. Although these preferences were not directly explored in this study, prior qualitative work using interviews of surgical and medical oncologists about their management preferences for SLN+ patients described concerns from some participants about the quality of nodal ultrasounds and radiologist awareness of MSLT-II protocols.^17^

Because patients receiving adjuvant immunotherapy are frequently surveilled with cross-sectional imaging by their medical oncologists, we were not surprised to observe similarities in the rates of adjuvant systemic therapy use and combined-modality adherence at many of the participating centers. Although the discussion of US versus cross-sectional surveillance modalities was beyond the scope of this study, future work should explore the utility of concomitant US and CT or PET and evaluate opportunities for improved coordination between medical and surgical oncologists for SLN+ patients receiving adjuvant immunotherapy. Ultimately, understanding and addressing barriers to surveillance adherence for any modality are important because approximately 10–20% of SLN+ patients undergoing nodal surveillance will experience a nodal recurrence within 2 years after SLNB and require timely intervention.^1,18,19^

Although no statistically significant differences in US adherence or combined-modality adherence were observed among the individual- and area-level SDoH variables studied in this cohort, the increased likelihood of LTFU among Medicaid and uninsured patients suggests that melanoma nodal surveillance, like the surveillance of many other cancer types previously studied, is sensitive to SDoH effects. Given the wide variation in geographic region, social vulnerability, insurance payor mix, and travel distances among participating centers in this study, this heterogeneity also may have affected which studied SDoH measures were more influential at a given center, highlighting the importance of local institutional efforts to measure and address social risk factors within their specific patient populations. For example, an English-speaking Medicare patient from a rural area with limited health literacy may face different barriers than a non-English-speaking underinsured patient who lives in an urban center with reliable public transportation, and strategies to address these different social needs should be tailored appropriately.

Across other cancer types, some examples of social risk factor mitigation strategies include patient navigation programs that offer services such as transportation assistance, vouchers to low-cost health food, access to trained interpreters, financial assistance including aid with completion of insurance forms, and care coordination.^9,20,21^ Although the evaluation of other SDoH factors was limited in this study due to its retrospective design, future prospective study of individual-level SDoH among melanoma nodal surveillance patients using validated SDoH assessments such as the Protocol for Responding to and Assessing Patient Assets, Risks, and Experiences (“PRAPARE”) screening tool would provide greater specificity for studying potential mediating social risk factors for nodal surveillance adherence.^22^ From a clinical standpoint for melanoma surgical oncologists, committing to patient-centered discussions with their SLN+ patients to convey the importance of regular surveillance visits will remain important. Furthermore, an understanding of an individual patient’s risk of nonadherence or LTFU may enable surgical oncologists to tailor their discussions with patients about the choice between nodal surveillance and CLND.

This study had several limitations that may affect its interpretation. First, the study period was an early post-MSLT-II period. Therefore, the heterogeneity among centers and overall lower US adherence rates observed in this study likely reflect an initial implementation process as these new nodal surveillance practices were developed across institutions. Whereas melanoma centers that were participating sites for MSLT-II may have derived some implementation benefits from prior exposure to nodal US protocols of the trial, we did not observe an increased likelihood of US adherence for patients treated at a prior MSLT-II site in the adjusted analysis.

Second, all the participating centers in this study were large academic cancer centers in the eastern half of the United States, which may limit the generalizability of these results to smaller cancer centers or those in other geographic regions because the social needs or other barriers to surveillance adherence may vary in these settings.

A third limitation was the missing CT and PET surveillance imaging data that were unavailable at the time of analysis for one participating site, which may have affected the combined-modality adherence model results as well as the potential for missing data related to surveillance imaging completed at outside facilities but not available to the participating center at the time of data abstraction.

Finally, although the focus of this study was on evaluating potential patient-level barriers to nodal surveillance, we recognize that surgeon- and institution-specific factors such as surveillance imaging preferences, adjuvant systemic therapy practices, and US availability and reliability could have a significant impact on surveillance adherence and warrant further study moving forward.

## Conclusion

Less than half of the SLN+ melanoma patients in this multi-institutional cohort achieved nodal surveillance US adherence, although rates improved with combined modalities that included cross-sectional imaging. Medicaid or lack of insurance were identified as social risk factors for loss to follow-up evaluation. As the population of SLN+ patients undergoing nodal surveillance continues to expand, optimizing surveillance practices among socially vulnerable populations, including quantifying and mitigating social risk factors, will be crucial to the ongoing implementation of nodal surveillance in clinical practice.

## Supplementary Information

Below is the link to the electronic supplementary material.

Supplementary file1 (DOCX 16 kb)

Supplementary file2 (DOCX 24 kb)
