# Supplementary material for: Impact of Social Determinants of Health on Melanoma Nodal Surveillance in a Multi-institutional Cohort
Source: Ann Surg Oncol. Author manuscript; Available in PMC 2025 Mar 1. (PMC11811232; doi:10.1245/s10434-024-16498-w)
Supplement: Supplemental File 1 [file NIHMS2049188-supplement-Supplemental_File_1.docx]

**Supplemental Table 1.** Participating institutions and locations.

| **Institution** | **Location** |
| --- | --- |
| University of Alabama at Birmingham (*Coordinating Center*) | Birmingham, AL, USA |
| Duke University Medical Center | Durham, NC, USA |
| Mayo Clinic | Rochester, MN, USA |
| Moffitt Cancer Center | Tampa, FL, USA |
| New York University - Langone Health | New York, NY, USA |
| University of Kentucky | Lexington, KY, USA |
| University of Michigan | Ann Arbor, MI, USA |
| University of North Carolina | Chapel Hill, NC, USA |
| University of Vermont | Burlington, VT, USA |
