# Supplementary material for: Impact of Social Determinants of Health on Melanoma Nodal Surveillance in a Multi-institutional Cohort
Source: Ann Surg Oncol. Author manuscript; Available in PMC 2025 Mar 1. (PMC11811232; doi:10.1245/s10434-024-16498-w)
Supplement: Supplemental File 2 [file NIHMS2049188-supplement-Supplemental_File_2.docx]

**Supplemental Table 2.** Multivariable logistic regression models evaluating likelihood of surveillance US adherence, stratified by SVI theme.

|  | **SVI Theme 1***  Socioeconomic Status | | | **SVI Theme 2***  Household Characteristics | | | **SVI Theme 3***  Racial & Ethnic Minority Status | | | **SVI Theme 4***  Housing Type & Transportation | | |
| --- | --- | --- | --- | --- | --- | --- | --- | --- | --- | --- | --- | --- |
| **Characteristic** [*Referent Value*] | **OR** | **95% CI** | **p-value** | **OR** | **95% CI** | **p-value** | **OR** | **95% CI** | **p-value** | **OR** | **95% CI** | **p-value** |
| **Age** (years) | 0.99 | 0.97 – 1.01 | 0.2 | 0.99 | 0.97 – 1.01 | 0.15 | 0.99 | 0.97 – 1.01 | 0.2 | 0.99 | 0.97 – 1.01 | 0.2 |
| **Male Sex** [*Female*] | 0.82 | 0.54 – 1.25 | 0.4 | 0.77 | 0.50 – 1.18 | 0.2 | 0.82 | 0.54 – 1.25 | 0.4 | 0.79 | 0.52 – 1.20 | 0.3 |
| **Travel Distance** [*<50 miles*]  50-100 miles | 1.20 | 0.71 – 2.01 | 0.5 | 1.23 | 0.72 – 2.08 | 0.4 | 1.24 | 0.74 – 2.07 | 0.4 | 1.22 | 0.73 – 2.04 | 0.4 |
| 100 miles | 0.88 | 0.53 – 1.48 | 0.6 | 0.93 | 0.55 – 1.57 | 0.8 | 0.93 | 0.55 – 1.56 | 0.8 | 0.93 | 0.55 – 1.55 | 0.8 |
| I**nsurance** [*Private*]  Medicaid | 0.45 | 0.15 – 1.38 | 0.2 | 0.49 | 0.16 – 1.52 | 0.2 | 0.49 | 0.16 – 1.47 | 0.2 | 0.43 | 0.14 – 1.34 | 0.15 |
| Medicare | 1.25 | 0.68 – 2.30 | 0.5 | 1.37 | 0.74 – 2.54 | 0.3 | 1.18 | 0.64 – 2.16 | 0.6 | 1.17 | 0.63 – 2.15 | 0.6 |
| Other Government | 0.81 | 0.13 – 4.97 | 0.8 | 1.08 | 0.17 – 6.68 | 0.9 | 0.67 | 0.11 – 4.04 | 0.7 | 0.72 | 0.11 – 4.56 | 0.7 |
| Not Insured | 0.48 | 0.14 – 1.70 | 0.3 | 0.57 | 0.16 – 2.05 | 0.4 | 0.51 | 0.14 – 1.82 | 0.3 | 0.42 | 0.12 – 1.52 | 0.2 |
| Unknown | 2.42 | 0.17 – 33.61 | 0.5 | 2.77 | 0.20 – 38.14 | 0.4 | 2.51 | 0.18 – 34.99 | 0.5 | 2.62 | 0.18 – 38.72 | 0.5 |
| **Received Adjuvant Systemic Therapy** [*No*] | 0.63 | 0.40 – 1.00 | 0.052 | 0.63 | 0.40 – 1.00 | 0.051 | 0.63 | 0.40 – 1.00 | 0.049 | 0.63 | 0.40 – 1.00 | 0.051 |
| **Prior MSLT-II Site** [*No*] | 0.86 | 0.15 – 4.84 | 0.9 | 0.90 | 0.15 – 5.21 | 0.9 | 0.87 | 0.15 – 4.95 | 0.9 | 0.82 | 0.15 – 4.47 | 0.8 |
| **SVI Theme 1** [*Lowest Quartile, <0.25*]  Mod Low Quartile [0.25 - 0.5] | 0.77 | 0.42 – 1.42 | 0.4 |  |  |  |  |  |  |  |  |  |
| Mod High Quartile [0.5 - 0.75] | 0.71 | 0.38 – 1.31 | 0.3 |  |  |  |  |  |  |  |  |  |
| Highest Quartile [>0.75] | 1.00 | 0.53 – 1.90 | >0.9 |  |  |  |  |  |  |  |  |  |
| **SVI Theme 2** [*Lowest Quartile, <0.25*]  Mod Low Quartile [0.25 - 0.5] |  |  |  | 0.67 | 0.36 – 1.23 | 0.2 |  |  |  |  |  |  |
| Mod High Quartile [0.5 - 0.75] |  |  |  | 0.40 | 0.21 – 0.76 | 0.005 |  |  |  |  |  |  |
| Highest Quartile [>0.75] |  |  |  | 0.77 | 0.41 – 1.45 | 0.4 |  |  |  |  |  |  |
| **SVI Theme 3** [*Lowest Quartile, <0.25*]  Mod Low Quartile [0.25 - 0.5] |  |  |  |  |  |  | 0.76 | 0.41 – 1.39 | 0.4 |  |  |  |
| Mod High Quartile [0.5 - 0.75] |  |  |  |  |  |  | 1.28 | 0.68 – 2.43 | 0.4 |  |  |  |
| Highest Quartile [>0.75] |  |  |  |  |  |  | 1.17 | 0.59 – 2.31 | 0.7 |  |  |  |
| **SVI Theme 4** [*Lowest Quartile, <0.25*]  Mod Low Quartile [0.25 - 0.5] |  |  |  |  |  |  |  |  |  | 0.71 | 0.39 – 1.26 | 0.2 |
| Mod High Quartile [0.5 - 0.75] |  |  |  |  |  |  |  |  |  | 1.14 | 0.63 – 2.07 | 0.7 |
| Highest Quartile [>0.75] |  |  |  |  |  |  |  |  |  | 0.60 | 0.32 – 1.12 | 0.11 |

**Note***:** Intraclass correlation coefficients were 0.27 for SVI Theme 1, 0.28 for SVI Theme 2, 0.28 for SVI Theme 3, and 0.28 for SVI Theme 4.
